# Predictors of Difficulty in Medication Intake in Europe: a Cross-country Analysis Based on SHARE

**DOI:** 10.14336/AD.2015.0925

**Published:** 2016-05-27

**Authors:** Daniela Figueiredo, Laetitia Teixeira, Veronica Poveda, Constança Paúl, Alice Santos-Silva, Elísio Costa

**Affiliations:** ^1^University of Aveiro, School of Health Sciences, Aveiro, Portugal.; ^2^Center for Health Technology and Services Research (CINTESIS.UA), Aveiro, Portugal.; ^3^Institute of Biomedical Sciences Abel Salazar, University of Porto, Porto, Portugal.; ^4^CINTESIS, Center for Health Technology and Services Research (CINTESIS), Porto, Portugal.; ^5^UCIBIO, REQUIMTE and Faculty of Pharmacy, University of Porto, Porto, Portugal.

**Keywords:** difficulty in take medication, elderly, SHARE, non-adherence, persistence

## Abstract

The aim of this study is to evaluate the prevalence and the predictors of difficulty in medication intake across Europe, using a cross-sectional design. We used data from all participants in the wave 4 of the SHARE (Survey of Health, Ageing, and Retirement in Europe) database, which is a cross national European survey. The difficulty in take medication was evaluated using an item from the “Limitations with activities of daily living”. Clinical and sociodemographic variables were evaluated as potential predictors. A total of 58 124 individual have been included in this work (mean age=64.9 ± 10.4 years; 43.3% male). The rate of difficulty in taking medication across the 16 European evaluated countries was 2.1%, presenting Spain the highest rate (5.7%) and Switzerland the lowest (0.6%). Increasing age, physical inactivity, physical limitations (mobility, arms function and fine motor limitations, and difficulties in picking up a small coin from a table), a poor sense of meaning in life, and losses in memory and concentration are independent and significant variables associated with difficulty in medication intake across Europe. Predictors of difficulties in medication intake are multicausal, including factors related to physical, cognitive and psychological conditions. Interventions aiming to optimize adherence to medication, particularly in elderly population, need to consider this diversity of determinants.

Older adults are the most rapidly growing segment of the population worldwide. In the European Union (EU), the number of people with 65 years of age or more will almost double over the next 50 years, from 85 million in 2008 to 151 million in 2060 (http://ec.europa.eu/research/innovation-union/). Though the percentage of healthy older adults has increased, it has been reported that more than 80% of the older population has at least one chronic disease and 50% has at least two [1].

Medications are often used as the primary approach to prevent and effectively manage chronic conditions. Evidence has shown that adherence to medication prescription improves health outcomes, reduce hospital admissions and prevent earlier mortality [2,3]. However, estimate rates of adherence to long-term medication regimen are of about 50%, and there is no evidence for significant changes in the past 50 years [4,5]. Non-adherence of the patient to the medication has been defined as taking less than 80% of the prescribed doses, or as exceeding the prescribed doses [5]. The consequences of non-adherence include poor clinical outcomes, increased morbidity and mortality, and unnecessary health care costs [6,7].

The factors contributing to non-adherence are multifaceted and embrace those that are related to patients (e.g., suboptimal health literacy, lack of social support, poor mental health), to physicians (e.g., prescription of complex drug regimens, inadequate communication), and to health care systems (e.g., limited access to health care) [8]. Usually age, itself, is not considered a predictor of non-adherence. Yet, the prevalence of risk factors for non-adherence increases with age [9]. Cognitive, sensorial and functional decline, poor social support, anxiety, depression symptomatology and reduced health literacy, have been linked to medication non-adherence in elderly patients [9-12]. Moreover, a number of chronic co-morbidities that often requires the co-prescription of multiple drugs, several dosage forms and frequent administration timing, are highly prevalent in older persons [11,13,14].

Older people are the major recipients of prescribed medications with an average of 3.6-fold more prescriptions than young adults [15]. About two-thirds of community-dwelling people aged 60 years or more take four or more medicines per day [9]. There is also a significant subgroup among the older population (frail older people with multiple chronic co-morbidities and functional impairments) who take an average of nine drugs per day [9]. Medication adherence/intake is, therefore, a complex and demanding issue, particularly in older people, whose frailty state might undermine their capacity to take medicines according to medical prescription.

In that way, the aims of this study was to evaluate several socio-economic and health-related factors as predictors of difficulty in taking medication across 16 European countries.

## MATERIAL AND METHODS

The Survey of Health, Ageing, and Retirement in Europe (SHARE) [16], is a multidisciplinary and cross-national panel database, which collects information on nationally representative samples of community-based population from 20 European countries (+Israel). In this work, we used data from wave 4 of this survey, which collected information about health, socio-economic status and social and family networks of individuals from 16 European countries (Austria, Belgium, Czech, Denmark, Estonia, France, Germany, Hungary, Italy, Netherlands, Poland, Portugal, Slovenia, Spain, Sweden and Switzerland). This wave of the SHARE survey (2011) contains 58,489 observations from individuals aged between 24 and 111 (in 2010). The target population of this survey was non-institutionalized individuals aged 50 years and older. The interviewees who have less than 50 years are spouses of the target population.

Data was collected by a comprehensive computer-assisted personal interview, which lasted for about 90 minutes, and by a supplementary paper Drop-Off questionnaire. In the computer-assisted interviews, the interviewers read the questions to the participants and typed their answers; the paper Drop-Off questionnaires were completed by the participants and returned at a later date. Informed consent was obtained from all participants prior to the interview [17].

### Evaluation of the difficulty in medication intake

The difficulty in medication intake was evaluated using one of the items from “Limitations with activities of daily living”. The question: “Please tell me if you have any difficulty with these activities because of a physical, mental, emotional or memory problem”, among a list of 13 activities that could be selected by the individuals, we selected for our study the response referring difficulty in “taking medication”. These responses generated, therefore, a dichotomic variable, according to individual’s selection, difficulty in taking medications or no selection of this option.

### Explanatory variables

The richness of the information in SHARE project allowed us to include a large number of potential explanatory variables, namely demographics (age and gender), education (“highest educational degree obtained”), subjective well-being (“future looks good”, “how satisfied with life”, “life has meaning” and “interest - part of EURO-D”), social support (“network satisfaction”, “given help last twelve months” and “received help from others”), depression (“depression scale EURO-D”), functional limitations (“difficulties in walking 100 meters”, “difficulties in picking up a small coin from a table”, “mobility, arm function and fine motor limitations” and “physical inactivity”), number of meals a day (“how many meals a day”), memory, number of chronic diseases, sleep (part of EURO-D), concentration (part of EURO-D) and enjoyment (part of EURO-D).

Age variable, was calculated according the response to date of birth and for the year 2010. Gender response generated a dichotomic variable, male or female. Education variable given by the response to the question “What is the highest school leaving certificate or school degree that you have obtained”, with the followed response options “Comprehensive school”, “Grammar school (not fee-paying)”, “Fee-paying grammar school”, “Sixth form College/Tertiary College”, “Public or other private school”, “Elementary school”, “Secondary modern/secondary school”, “Technical school (not college)”, “No degree yet/still in school”, “None” and “Other type (also abroad)”, has been analyzed as a continuous variable.

The variable “future looks good” derivate from the response to the question “How often do you feel that the future looks good for you?”, which had four response options “often”, “sometimes”, “rarely” or “never”.

The variable “how satisfied with life” derived from the response to the question “On a scale from 0 to 10 where 0 means completely dissatisfied and 10 means completely satisfied, how satisfied are you with your life?”, has been analyzed as a continuous variable.

The variable “life has meaning” derived from the question “How often do you feel that your life has meaning?” with four response options “often”, “sometimes”, “rarely” or “never”.

Social support has been evaluated using three variables “network satisfaction”, “given help last twelve months” and “received help from others”. The results of these variables were in accordance with the responses to the following questions: “Overall, how satisfied are you with the [relationship that you have with the person/relationships that you have with the persons] we have just talked about? Please answer on a scale from 0 to 10 where 0 means completely dissatisfied and 10 means completely satisfied”; “In the last twelve months, have you personally given personal care or practical household help to a family member living outside your household, a friend or neighbour?” and “Thinking about the last twelve months has any family member from outside the household, any friend or neighbour given you [or] [your] [husband/wife/partner/partner] personal care or practical household help?”, respectively. The first variable (network satisfaction) has been considered as a continuous variable and the other two (given help last twelve months and received help from others) as dichotomic variables (Yes or No).

Functional limitations associated variables that have been included, were “difficulties in walking 100 meters”, “difficulties in picking up a small coin from a table”, “mobility, arm function and fine motor limitations” and “physical inactivity”. The first two variables result from the question “We need to understand difficulties that people may have with various activities because of a health or physical problem. Please tell me whether you have any difficulty doing each of the everyday activities on card 10. Exclude any difficulties that you expect to last less than three months”. Mobility (number of limitations with mobility, arm function & fine motor function) variable is based on the response of 10 different question items, resulting in a score evaluated as a continuous variable. The score for these continuous variables corresponds to the number of limitations with mobility, arm function & fine motor function reported by each individual. The physical inactivity variable is constructed on the basis of the questions “We would like to know about the type and amount of physical activity you do in your daily life. How often do you engage in vigorous physical activity, such as sports, heavy housework, or a job that involves physical labor?” and “How often do you engage in activities that require a moderate level of energy such as gardening, cleaning the car, or doing a walk?”, regarding levels of vigorous and moderate physical activity, respectively. Physical inactivity is defined as never or almost never engaging in neither moderate nor vigorous physical activity.

The variable “number of meals a day” results from the response to the question “How many full meals a day do you usually eat?”. This variable has been evaluated as continuous.

The variable “memory” results from the question “How would you rate your memory at the present time? Would you say it is excellent, very good, good, fair or poor?” with five possible responses: excellent, very good, good, fair or poor, and has been evaluated as discrete variable.

The variable “number of chronic diseases” is based on the number of chronic diseases reported by each individual.

The variables “interest”, “sleep”, “concentration” and “enjoyment” are different domains of EURO-D scale. Moreover, “depression” is the total score of the EURO-D scale, which has been included in SHARE database.

### Statistical analysis

Descriptive analysis was performed in order to obtain an estimate of the proportion of individuals with difficulty in medication intake across 16 European countries. Given the multilevel structure of data, with individuals nested in each country, a multilevel logistic regression approach was used, considering the difficulty in medication intake as the dependent variable. In a first step, multilevel univariable logistic regression models were performed, considering each covariate, to identify potential factors associated with the outcome variable. Significant covariates retained in the first step were included in a multilevel multivariate logistic regression model. The final model was composed only by significant covariates, considering a backward selection method. Odds ratios (OR) and their 95% confidence intervals (CI) are reported. Analyses were performed using IBM SPSS (version 21) and a significance level of 0.05 was considered.

## RESULTS

From the total of 58 489 individuals that have participated in SHARE survey, 0.6% (365) of them did not give information about the difficulty, or not, in medications intake. Therefore, this work included only 58 124 individuals with a mean (±SD) age of 64.9 ± 10.4 years; 43.3% (25 193) of them were male. Considering the large sample size, the percentage of missing values for the potential explanatory variables was considered as not relevant for the analysis of the results.

**Table 1:** Distribution of the individuals from each country presenting difficulties in medication intake, and in accordance with age (less or more than 65 years).

Country	All individuals (n)	Individuals with less than 65 years (n)	Individuals with more than 65 years (n)	Cases of difficulty in medication intake		
				All individuals n (%)	Individuals with less than 65 years n (%)	Individuals with more than 65 years n (%)
Austria	5241	2781	2456	68 (1.3)	8 (0.3)	60 (2.4)
Germany	1571	653	918	38 (2.4)	3 (0.5)	35 (3.8)
Sweden	1946	706	1240	54 (2.8)	1 (0.1)	53 (4.3)
Netherlands	2755	1503	1252	26 (0.9)	3 (0.2)	23 (1.8)
Spain	3555	1612	1943	203 (5.7)	30 (1.9)	173 (8.9)
Italy	3574	1736	1838	95 (2.7)	5 (0.3)	90 (4.9)
France	5747	3163	2581	104 (1.8)	16 (0.5)	88 (3.4)
Denmark	2269	1306	963	45 (2.0)	4 (0.3)	41 (4.3)
Switzerland	3739	2052	1687	21 (0.6)	2 (0.1)	19 (1.1)
Belgium	5276	3086	2189	108 (2.0)	18 (0.6)	90 (4.1)
Czechia	6079	3356	2719	100 (1.6)	28 (0.8)	72 (2.6)
Poland	1717	944	772	64 (3.7)	14 (1.5)	50 (6.5)
Hungary	3065	1773	1291	53 (1.7)	11 (0.6)	4 (3.3)
Portugal	2054	1152	898	46 (2.2)	9 (0.8)	37 (4.1)
Slovenia	2741	1528	1213	46 (1.7)	13 (0.9)	33 (2.7)
Estonia	6795	3280	3515	146 (2.1)	26 (0.8)	120 (3.4)
**Total**	**58124**	**30440**	**26449**	**1217 (2.1)**	**191 (0.6)**	**1026 (3.7)**

The rate of difficulty in medication intake across the 16 evaluated countries was 2.1%, presenting Spain the highest rate and Switzerland the lowest (Table 1). A significantly increased difficulty in medication intake was found in the individuals with 65 years or more, when compared with those with less than 65 years (3.7% *vs*. 0.6%, p<0.001).

Table 2 displays the association of the explanatory variables with the difficulty in medication intake using unadjusted models; we found that difficulty in medication intake is significantly associated with all evaluated explanatory variables, with exception of gender.

Using adjusted models, we found that increasing age [OR=0.441 (0.376-0.517)] and decreased mobility, arm function and fine motor limitations [OR= 1.074 (1.051-1.097)] are associated with difficulties in medication intake. We also found that individuals without difficulties in picking up a small coin from table [OR= 0.441 (0.376-0.517)], performing physical activity [OR= 0.822 (0.736-0.918)] and with no concentration difficulties [OR= 0.822 (0.736-0.918)] showed a lower difficulty in medication intake. Individuals that reported excellent [OR= 0.555 (0.440-0.701)], very good [OR= 0.546 (0.456-0.654)], good [OR= 0.531 (0.455-0.620)] or fair [OR= 0.530 (0.454-0.618)] memory showed a lower difficulty in medication intake, when compared with those presented poor memory. Moreover, individuals who reported that their own life has meaning often [OR= 0.781 (0.636-0.960)] and sometimes [OR= 0.751 (0.608-0.927)] showed a lower difficulty in medication intake, when compared with those who never feel that their life has meaning (Table III).

**Table 2:** Association of explanatory variables with difficulty in medication intake (unadjusted models)

	n	Cases of difficulty in take medication n (%)	Unadjusted		
			OR	CI 95%	p
**Difficulties in walking 100 meters**					
Yes	6986	846 (12.1)	1	-	-
No	51125	368 (0.7)	0.053	0.047-0.060	<0.001
**Difficulties in picking up a small coin from a table**					
Yes	2528	660 (26.1)	1	-	-
No	55583	554 (1.0)	0.043	0.038-0.049	<0.001
**How satisfied with life**	56843	827 (1.5)	0.713	0.692-0.734	<0.001
**Life has meaning**					
Never	1690	127 (7.5)	1	-	-
Rarely	4189	190 (4.5)	0.579	0.458-0.731	<0.001
Sometimes	13248	244 (1.8)	0.228	0.183-0.286	<0.001
Often	37407	254 (0.7)	0.086	0.06-0.108	<0.001
**Future looks good**					
Never	4754	317 (6.7)	1	-	-
Rarely	11034	236 (2.1)	0.286	0.240-0.341	<0.001
Sometimes	20335	170 (0.8)	0.108	0.089-0.131	<0.001
Often	20171	87 (0.4)	0.055	0.043-0.071	<0.001
**How many meals a day**	57661	1086 (1.9)	1.103	1.026-1.185	0.008
**Memory**					
Poor	3900	470 (12.1)	1	-	-
Fair	11396	259 (2.3)	0.122	0.105-0.143	<0.001
Good	25322	176 (0.7)	0.044	0.037-0.053	<0.001
Very good	13370	37 (0.3)	0.020	0.015-0.029	<0.001
Excellent	3324	17 (0.5)	0.028	0.017-0.045	<0.001
**Highest educational degree obtained**	37758	684 (1.8)	1.009	1.006-1.012	<0.001
**Gender**					
Female	32931	701 (2.1)	1	-	-
Male	25193	516 (2.0)	0.953	0.849-1.069	0.411
**Network satisfaction**	54707	849 (1.6)	0.902	0.860-0.946	<0.001
**Received help from others**					
No	31799	348 (1.1)	1	-	-
Yes	8119	433 (5.3)	5.547	4.815-6.452	<0.001
**Given help last twelve months**					
No	29472	733 (2.5)	1	-	-
Yes	10446	50 (0.5)	0.196	0.147-0.262	<0.001
**Number of chronic diseases**	58074	1214 (2.1)	1.535	1.492-1.580	<0.001
**Mobility, arm function and fine motor limitations**	58111	1214 (2.1)	1.774	1.736-1.814	<0.001
**Physical inactivity**					
Never vigorous nor moderate physical activity	7188	778 (10.8)	1	-	-
Other	50482	312 (0.6)	0.054	0.047-0.062	<0.001
**Sleep (part of EURO-D)**					
Selected	20697	518 (2.5)	1	-	-
Not selected	36634	451 (1.2)	0.497	0.437-0.565	<0.001
**Interest (part of EURO-D)**					
Selected	5356	362 (6.8)	1	-	-
Not selected	51909	576 (1.1)	0.167	0.146-0.192	<0.001
**Concentration (part of EURO-D)**					
Selected	10826	661 (6.1)	1	-	-
Not selected	46374	287 (0.6)	0.098	0.085-0.112	<0.001
**Enjoyment (part of EURO-D)**					
Selected	8115	417 (5.1)	1	-	-
Not selected	49135	516 (1.1)	0.198	0.173-0.226	
**Depression scale EURO-D - high is depressed**	56527	838 (1.5)	1.469	1.434-1.504	<0.001
**Age**	58107	1217 (2.1)	1.118	1.111-1.124	<0.001

**Table 3:** Association of explanatory variables with difficulty in medication intake (adjusted model)

	n	Cases of difficulty in take medication n (%)	Adjusted		
			OR	CI 95%	p
**Difficulties in picking up a small coin from a table**					
Yes	2528	660 (26.1)	1	-	-
No	55583	554 (1.0)	0.441	0.376-0.517	<0.005
**Life has meaning**					
Never	1690	127 (7.5)	1	-	-
Rarely	4189	190 (4.5)	0.835	0.663-1.051	0.125
Sometimes	13248	244 (1.8)	0.751	0.608-0.927	0.008
Often	37407	254 (0.7)	0.781	0.636-0.960	0.019
**Memory**					
Poor	3900	470 (12.1)	1	-	-
Fair	11396	259 (2.3)	0.530	0.454-0.618	<0.001
Good	25322	176 (0.7)	0.531	0.455-0.620	<0.001
Very good	13370	37 (0.3)	0.546	0.456-0.654	<0.001
Excellent	3324	17 (0.5)	0.555	0.440-0.701	<0.001
**Mobility, arm function and fine motor limitations**	58111	1214 (2.1)	1.074	1.051-1.097	<0.001
**Physical inactivity**					
Never vigorous nor moderate physical activity	7188	778 (10.8)	1	-	-
Other	50482	312 (0.6)	0.666	0.588-0.755	<0.001
**Concentration (part of EURO-D)**					
Selected	10826	661 (6.1)	1	-	-
Not selected	46374	287 (0.6)	0.822	0.736-0.918	<0.001
**Age**	58107	1217 (2.1)	1.005	1.001-1.010	0.023

## DISCUSSION

Medication adherence and persistence is recognized as a worldwide public health problem, with important implications for the management of chronic diseases. Non-adherence to medical plans is found in every levels of the population, particularly in older adults, due to the high number of co-existing diseases and consequent polypharmacy. Indeed, older people often experience problems in daily living, because of chronic illnesses or health-related disabilities. These difficulties restrict their ability to perform self-care, which is a common reason why older people seek help from outsiders, move to assisted living communities, or enter nursing homes.

Difficulty in medication intake associated with non-adherence and persistence in old persons, can be related with several factors, such as physical, mental, emotional or memory problems [18,19]. In this study, we evaluated the prevalence of difficulty in medication intake and it’s predictors across Europe.

The overall findings suggested that age, physical limitations, poor sense of meaning in life, and memory and concentration limitations, are predictors of difficulty in medication intake and, therefore, in risk of poor adherence to medication.

Globally, the prevalence of difficulty in medication intake is 2.1% across the European countries. This prevalence was significantly higher in individuals with more than 65 years old (3.7%), when compared with those with less than 65 years old (0.6%). Indeed, we found that increasing age is a predictor of difficulty in medication intake. Considering the number of individuals with more than 65 years in Europe in 2013 (93 084 401) (www.prodata.pt), we can assume that there are about 3 444 123 individuals over 65 years old who have difficulties in medication intake in Europe. These individuals are at risk of non-adherence to medication, which is known to have an important impact in health care costs, quality of life and health outcomes [6,20].

Functional decline, in terms of mobility, arms function and fine motor limitations, physical inactivity and difficulties in picking up a small coin from a table, was also a significant predictor of difficulties in medication intake. These findings are in line with past reports suggesting that older adults with functional limitations might have difficulties in some actions that are important for medication management [21,22]. Indeed, medication management requires functional capacities to coordinate important tasks, including opening safety-capped vials, removing tablets from vials or differentiating tablets colors [23]. Our findings stress the need to analyze mobility and fine motor limitations as significant risk factors for medication non-adherence. Although several older people who have these conditions might receive support from family with medication management, many others live alone and need to self-administer medication. This later group is likely to be at higher risk for medication non-adherence and might benefit from assessment and personalized interventions concerning medication management [23].

Past studies have shown the links of meaning in life with health and mortality [24-26]; however much less is known about its relation to medication (none) adherence. Our study helps to shed some light on this issue, as a poor sense of meaning in life has emerged as significant predictor of difficulties in medication intake. A rational to explain this link is that a sense of meaning in life might act as a motivation to maintain one’s physical and mental health [26]. In other words, older people who do not have a strong meaning in life are less likely to engage in beneficial health behaviors. Another explanation is that the absence of meaning in life may be affected by a poor health status, which indirectly influences medication intake. Nevertheless, the mediational pathway of life meaning in medication intake remains clearly understudied and further investigation on this issue is warranted.

Consistent with previous research [10,12], cognitive decline, showed by diminished memory and concentration capacity, was also found as a predictor of difficulty in medication intake among older people. As stated by Insel et al. [10], medication adherence involves the role of the executive function, as taking medication requires developing and implementing a plan to adhere, remembering to adhere and remembering whether the medicine was taken as desired. It also involves working memory, as a person must keep the intention to take medication, while doing other things, like checking the time, the dosage, pouring a glass of water and getting the medicine. Furthermore, medication adherence might depend on the encoding and storage information about the medicine (regimen, dosage), demands concentration capacity. Therefore, the current findings highlight the importance of analyzing cognitive functions – concentration and memory - as risk factors for difficulties in medication intake. Consequently, helping older people remember to take their medication at correct doses must be a priority to avoid negative health outcomes and unnecessary expenditures. Reminder-based interventions tailored to older people using short message services from mobile phones might be a simple and cost-effective way of improving medication intake [27,28].

Some limitations of the current study need to be addressed. First, the medication regimen of the respondents was unknown; thus, further studies with older people taking multiple medications are needed to confirm risk factors. Second, it is well known that people who volunteer to participate in research surveys like SHARE are likely to be more motivated and healthier [29]. Thus, older people with multiple chronic conditions and a complex poly-pharmacy regimen might have been excluded. Third, medication adherence was not the primary outcome of the SHARE survey and difficulty in taking medication was assessed with a single question. Well designed survey studies with different measures of medication adherence are therefore needed to confirm the extent of the current findings.

In conclusion, predictors of difficulties in medication intake are multifactorial, including determinants related to physical, cognitive and psychological conditions. Such predictive factors may help to identify medication non-adherence among older people and, ultimately, to decrease mortality risk. Therefore, interventions aiming to optimize medication adherence need to be tailored and multifaceted, as no single strategy will be effective in all older people groups.
